# Pneumoperitoneum in Veterinary Laparoscopy: A Review

**DOI:** 10.3390/vetsci7020064

**Published:** 2020-05-12

**Authors:** Jacqueline Scott, Ameet Singh, Alexander Valverde

**Affiliations:** 1 College of Veterinary Medicine, University of Illinois, Urbana-Champaign, IL 61802, USA; 2 Department of Clinical Studies, Ontario Veterinary College, University of Guelph, Guelph, ON N1G 2W1, Canada; amsingh@uoguelph.ca (A.S.); valverde@uoguelph.ca (A.V.)

**Keywords:** pneumoperitoneum, laparoscopy, capnoperitoneum, carbon dioxide, insufflation

## Abstract

*Objective:* To review the effects of carbon dioxide pneumoperitoneum during laparoscopy, evaluate alternative techniques to establishing a working space and compare this to current recommendations in veterinary surgery. *Study Design:* Literature review. *Sample Population:* 92 peer-reviewed articles. *Methods:* An electronic database search identified human and veterinary literature on the effects of pneumoperitoneum (carbon dioxide insufflation for laparoscopy) and alternatives with a focus on adaptation to the veterinary field. *Results:* Laparoscopy is the preferred surgical approach for many human and several veterinary procedures due to the lower morbidity associated with minimally invasive surgery, compared to laparotomy. The establishment of a pneumoperitoneum with a gas most commonly facilitates a working space. Carbon dioxide is the preferred gas for insufflation as it is inert, inexpensive, noncombustible, colorless, excreted by the lungs and highly soluble in water. Detrimental side effects such as acidosis, hypercapnia, reduction in cardiac output, decreased pulmonary compliance, hypothermia and post-operative pain have been associated with a pneumoperitoneum established with CO_2_ insufflation. As such alternatives have been suggested such as helium, nitrous oxide, warmed and humidified carbon dioxide and gasless laparoscopy. None of these alternatives have found a consistent benefit over standard carbon dioxide insufflation. *Conclusions:* The physiologic alterations seen with CO_2_ insufflation at the current recommended intra-abdominal pressures are mild and of transient duration. *Clinical Significance:* The current recommendations in veterinary laparoscopy for a pneumoperitoneum using carbon dioxide appear to be safe and effective.

## 1. Introduction

The first described use of a light source to visualize a human orifice was in 1805 by Philip Bozzini to visualize the urethra and urinary bladder [1]. In 1853 this was further developed by Desormeaux into a rudimentary endoscope however, this device was wrought with thermal injury complications [1]. The first reported use of endoscopy examination of the peritoneal cavity (laparoscopy) came 1901 by Dr. Kelling, using oxygen to create a pneumoperitoneum [1]. It was not until 1924 that carbon dioxide (CO_2_) was proposed as the preferred gas for insufflation [1]. Modern laparoscopy was pioneered by gynecologists in the 1960s and 1970s, however it was not until the 1980s that laparoscopic surgery started to be more widely accepted [1]. Laparoscopic surgery has been found by multiple systematic reviews to reduce postoperative discomfort and shorten hospitalization times in people [2,3,4]. Today, laparoscopic surgery is considered the gold standard for many procedures, with over 500,000 laparoscopic cholecystectomies performed annually in the United States alone [5].

Laparoscopy involves establishing a pneumoperitoneum, which is defined as the insufflation of gas into the abdomen [6]. This creates a working space and allows for a camera and other instruments to be introduced into the abdomen through small incisions in order to perform minimally invasive surgery [6]. A variety of gases have been used to produce this pneumoperitoneum including oxygen, helium and nitrous oxide, [1,7,8] however CO_2_ is the most commonly used gas because it is cheap, noncombustible, colorless, excreted by the lungs and highly soluble in water, reducing the risk of gas embolism [9]. A pneumoperitoneum established with CO_2_ is defined as a capnoperitoneum [6]. Carbon dioxide is transferred from gas tanks towards the patient through a mechanical insufflator via a hose, and introduced into the peritoneal cavity through a cannula [6]. The mechanical insufflator allows monitoring and maintenance of the intra-abdominal pressure (IAP) at recommended values, usually around 10 mm Hg [6]. This pressure provides adequate working space, while maintaining physiologically acceptable cardiorespiratory depression [10]. 

The development of minimally invasive surgery (MIS) in veterinary medicine parallels the human field, however, with a 20 year delay [11]. Initially developed as a diagnostic tool, the practice of minimally invasive surgery has grown rapidly in the last 15 years in veterinary medicine [12,13,14]. In a 2010 survey by Bleedorn et al., 1216 veterinary surgery diplomates were surveyed on their current MIS use in practice [15]. The results of the survey found eighty-six percent of small animal diplomates performed minimally invasive surgery with a steady increase in use over time documented. Less than 20 of the diplomates who qualified prior to 1985 were using MIS, compared to almost 120 who qualified in the last 4 years prior to 2010 [15]. 

Laparoscopy is indicated in a variety of small animal conditions, from organ biopsies, prophylactic gastropexy and ovariohysterectomy to more advanced procedures such as cholecystectomy, adrenalectomy or ureteronephrectomy [16]. Distinct advantages over a traditional “open” approach have been reported in the veterinary literature with a reduction in pain, [17] a more rapid return to function, [13] lower surgical site infection rate, [18] and reduced hospitalization time [19]. 

While CO_2_ is the most common gas used for insufflation, it has been found that a pneumoperitoneum established with CO_2_ can lead to numerous detrimental side effects on cellular, metabolic, cardiovascular and respiratory physiology [9,20]. The aim of this literature review was to assess the techniques used to create a working space during laparoscopy and compare this to current recommendations in veterinary laparoscopy.

## 2. Methods

A literature search was conducted through the Medline and PubMed database from 1975 to 2018 using the search terms laparoscopy, capnoperitoneum, pneumoperitoneum, veterinary, dog and cat. Articles were evaluated for relevance to the detrimental effects of CO_2_ insufflation and alternatives with a focus on adaptation to the veterinary field. Individual case reports were excluded; however, review articles, in vitro studies and experimental animal models were included.

## 3. Results

A literature search revealed 78 journal articles matching the required criteria. The majority (38) of the articles were human randomized clinical trials or review articles. Experimental animal models made up 14 of the articles, including pig (7), rat (5), mice (1) and dog (1) studies. The veterinary literature contributed 25 articles, including 15 dog, 3 cat and 7 studies including both dogs and cats. In addition, one horse study was evaluated. The duration of publication extends over a 47-year period from 1971 to 2018, with most of the literature published between 1990 and 2010.

### 3.1. Cardiorespiratory Alterations

Carbon dioxide insufflation in the peritoneum causes systemic effects through two mechanisms; first, the absorption through the peritoneal surface of CO_2_ into the circulation, which can produce systemic acidosis and hypercapnia, [21] leading to vasodilatory effects on the blood vessels, myocardial depression and indirect sympathetic stimulation [22]; and second, an increase in the IAP that leads to alterations in almost all cardiorespiratory variables including heart rate, cardiac output, stroke volume, inferior vena cava flow, systemic vascular resistance, arterial partial pressure of oxygen, oxygen delivery, mean arterial pressure, pulmonary compliance and minute ventilation [10,23,24]. While it is generally considered that these alterations are transient, their effects may be of importance in the cardiac or respiratory compromised patient [25]. 

PaCO_2_ and end-tidal CO_2_ (ETCO_2_) increase in a linear fashion following the initiation of a pneumoperitoneum, leading to a concurrent drop in arterial pH [24,26]. In order to prevent hypercapnia and acidosis, intermittent positive pressure ventilation (IPPV) is utilized to remove excessive CO_2_ [25]. In addition to the absorption of insufflated CO_2_, increases in IAP limit diaphragm excursion, reduce pulmonary compliance, functional residual capacity and vital capacity of the lung [10,24,25]. This also contributes to an increase in PaCO_2_ and ETCO_2_, and an inability to compensate for this elevation, which leads to fatigue and the need for mechanical ventilation [26].

Cardiovascular function is affected both negatively and positively by the increase in IAP from the pneumoperitoneum. The health status of the patient can often determine which effects become more relevant. An increase in IAP by pneumoperitoneum decreases venous return and end diastolic volume, due to compression of the vena cava, which reduces stroke volume, and potentially cardiac output if heart rate does not compensate for the changes in stroke volume [27]. A decrease of 15–80% has been reported in the research and clinical veterinary literature, with IAP ranging from 6–30 mm Hg [10,24]. 

When pneumoperitoneum is established at an IAP of ~10 mm Hg, cardiac output is often preserved despite a fall in stroke volume, due to a concurrent increase in heart rate, [10,24] and in some cases cardiac output is actually reported to increase, due to a displacement of splanchnic volume [28]. Mayhew et al. (2013) evaluated working space and cardiorespiratory variables at different insufflation pressures in cats (4, 8 and 15 mm Hg), [29] and found that no benefit in working space was gained above 8 mm Hg, while a significant increase in PaCO_2_ and a significant decrease in pH was noted at 15 mm Hg [29]. Other cardiovascular parameters, including heart rate, cardiac output, systemic vascular resistance, stroke volume, and mean arterial pressure were similar between the two higher insufflation pressures [29].

Systemic vascular resistance is reported to exhibit increasing trends to no change during laparoscopy [7,29,30]. Ho et al. (1995) found no significant increase in systemic vascular resistance in research pigs until intra-abdominal pressures of over 20 mm Hg were applied, [30] while Duke et al. (1996) and Mayhew et al. (2013) both reported a trend in dogs and cats, respectively, towards elevation [10,29].

Arterial blood pressure is expected to increase due to sympathetic stimulation, increases in cardiac output and increased IAP [28,29,30]. In cats, an insufflation pressure of 15 mm Hg resulted in higher mean arterial pressure than insufflation with 4 mm Hg [29]. Several studies have however, reported stable arterial blood pressure [7,20,31,32] in spite of insufflation pressures in dogs of 15 mm Hg [7,32] or 30 mm Hg [32], even in Trendelenburg and reverse Trendelenburg positions [33]. Blood pressure is often used as a convenient surrogate to blood flow, however cardiovascular studies have found no correlation between blood pressure and cardiac output [32]. Alterations in cardiac output and stroke volume were not accompanied by nor could they detect changes in mean arterial pressure in dogs undergoing pneumoperitoneum [33]. Cardiac output is an important variable to determine sufficient blood flow and oxygenation to vital organs, [34] but is rarely measured in clinical practice.

Anesthesia further exacerbates cardiorespiratory compromise and special consideration of anesthetic protocols in patients undergoing pneumoperitoneum is warranted [27]. In particular mechanical ventilation with close monitoring of ETCO_2_, blood pressure, and blood gas alterations is recommended. As such, laparoscopy in patients with neurological, respiratory, renal or cardiac compromise may be contraindicated [25]. 

Given these findings, the current veterinary literature supports CO_2_ insufflation to an IAP of 10 mm Hg for dogs and 8 mm Hg for cats [10,24,26,29]. 

### 3.2. Hypothermia

It has also been hypothesized that laparoscopic surgery using CO_2_ insufflation exacerbates perioperative hypothermia [35]. Heat production in the body is due to metabolism and is regulated by thermoreceptors in the hypothalamus that initiate reflexes to increase heat production and reduce heat loss [36]. Thermoregulation normally has a very narrow set point of +/− 0.2 °C, however anesthetic drugs can increase the interthreshold range by approximately 3.5 °C [37]. Surgery increases heat loss from radiation, conduction, convection and evaporation [27,37]. Radiation has been identified as the most important cause of perianesthestic heat loss and unfortunately is not inhibited by traditional methods to prevent hypothermia (warmed water blankets, forced warmed air heaters) [37]. 

Standard CO_2_ (STCO_2_) used for insufflation is room temperature or 22 °C with 0% relative humidity [35]. The mechanism explaining laparoscopic hypothermia is based on heat loss from convection and evaporative cooling. Convection is energy transfer from the warm abdominal cavity and is proportional to the rate of gas flow, specific heat capacity of the gas and the temperature of the gas used [38]. The average CO_2_ gas flow in a human patient varies considerably (12–801 L) and is related to the volume of CO_2_ leaking around port sites, the duration and type of procedure and the number of instrument changes [39]. Evaporation is heat loss from liquid water to saturate the insufflated CO_2_ particles [35] and is thought to contribute the most to heat loss during laparoscopy [38]. 

Inadvertent perianesthetic hypothermia is one of the most common complications during surgery [36] and has numerous detrimental effects leading to an increase in patient morbidity [36,40,41,42,43]. (Table 1)

The insufflation of a more physiological CO_2_ environment has also been suggested to minimize the detrimental side effects. The warming (37 °C) and humidification (98%) of CO_2_ (WHCO_2_), to more closely resemble the patients peritoneal cavity, is thought to preserve cellular and metabolic function, while also preventing hypothermia [45,46]. The proposed thermoregulatory benefits of altering the temperature and humidity of insufflated CO_2_ has been evaluated extensively for laparoscopic surgery in humans [47,48,49], however a recent meta-analysis by the Cochrane Collaboration found no significant benefit in the use of warmed CO_2_ insufflation with or without humidification compared to STCO_2_ [50]. Furthermore, a recent study in dogs found that mean core body temperature was significantly lower with WHCO_2_ compared to STCO_2_ [28].

Given these findings the current veterinary literature does not support warmed or humidified CO_2_ insufflation, but instead judicious use of traditional thermoregulatory modalities during laparoscopy such as warmed water blankets and forced warmed air heaters [28,50,51]. 

### 3.3. Peritoneal Irritation

The peritoneum is a serous membrane surface comprised of squamous epithelial cells and is easily injured [52]. Its primary function is to reduce friction within the abdominal cavity [52]. It has been found the insufflation of the peritoneal cavity with CO_2_ alters peritoneal morphology, pH and the local inflammatory response [26,53]. In the acute inflammatory response there is vasodilation, an increase in vascular permeability and leukocyte extravasation leading to an influx of inflammatory mediators and acute phase proteins [54]. Pro-inflammatory cytokines TNF-α, Il-1β, IL-6 are released from macrophages, which in turn alter the synthesis of acute phase proteins in the liver [54]. Positive acute phase proteins increase in plasma concentration by 25% during inflammation and include C-reactive protein, serum amyloid A, serum amyloid P, complement proteins, coagulation factors and kallikrein–kinin proteins [54]. These cytokines and acute phase proteins can be measured in the serum as biomarkers for inflammation [54,55]. 

The use of biomarkers in human medicine to evaluate the postoperative inflammation has proven minimally invasive surgery to have a reduced inflammatory response compared to traditional open techniques as measured by IL-6 and CRP [56]. In humans, laparoscopic cholecystectomy is a common procedure performed for symptomatic cholecystolithiasis [57]. 

Laparoscopic cholecystectomy has been associated with a significantly shorter hospital stay and shorter convalescence than open cholecystectomy [57]. In human patients undergoing laparoscopic cholecystectomy, peak IL-6 and CRP concentrations have been found to be significantly lower (51 pg/mL and 24 mg/L, respectively) than patients who underwent open cholecystectomy (124 pg/mL, 104 mg/L, respectively) [58]. Researchers concluded this finding supports the true minimally invasive nature of laparoscopic cholecystectomy [58]. 

Laparoscopic surgical stimulation and the sequential inflammatory response can lead to the formation of adhesions if vascular injury occurs [52]. The propensity for adhesion formation is species dependent and is a significant contributor to morbidity in human patients [52]. While it was shown by some authors that laparoscopy reduces adhesion formation, compared to laparotomy, it does not completely eliminate them, [59] and the impact of CO_2_ insufflation on adhesion formation is inconclusive [60]. 

When the peritoneum is exposed to CO_2_ gas, retraction of mesothelial cells is seen using scanning electron microscopy with exposure of the underlying basal lamina [61,62]. Mice exposed to a pneumoperitoneum at an IAP of 6 mm Hg for 30 min, [62] had no alterations in peritoneal morphology intra-operatively, however progressive changes with widening of intercellular clefts and exposure of the basal lamina was evident in the first 12 h postoperatively. Mesothelial regeneration was noted over the remaining 84 h and a nearly confluent layer of microvilli-covered cells were seen at 96 h postoperatively [62]. 

Healthy mature aged dogs exposed to STCO_2_ (22 °C and 0% relative humidity) for 90 min at an IAP of 10 mm Hg, have been found to develop mesothelial desquamation with exposure of the basal lamina compared to dogs exposed to WHCO_2_ (38 °C and 98% relative humidity) in which the peritoneum was relatively preserved [28]. Interestingly, the use of WHCO_2_ did not result in better postoperative analgesia in research dogs when compared to STCO_2_, despite more peritoneal irritation with the latter [28]. 

While an interesting area of further research, the current veterinary literature has not been able to demonstrate the significance of peritoneal irritation or find a reliable alternative, as such the current recommendation is for STCO_2_ insufflation [28]. 

### 3.4. Oncologic Considerations

Denuded basal lamina as seen following peritoneal exposure to STCO_2_, is thought to predispose the peritoneal surface to tumor cell implantation, [63] with CO_2_ insufflation also contributing to the risk of port site metastasis through tumor cell aerosolization [64]. Port site metastasis is reported in 1–4% of human patients undergoing laparoscopic surgery for malignant conditions [65]. It is most commonly seen in between 3–9 months after the original surgery, and presents as firm hard nodules over previous port sites [65]. 

In a rat study using scanning electron microscopy, tumor cell implantation onto damaged peritoneal surface with basal lamina exposure could be seen in less than 2 h, compared to rats with a normal peritoneal surface in which tumor cell implantation was rare [66]. 

Compared to an open laparotomy, patients exposed to STCO_2_ appear to be at a higher risk for incisional site metastasis [63,65]. Jacobi et al. investigated the incidence of incisional site tumors in rats exposed to STCO_2_, helium insufflation at an IAP of 8 mm Hg or room air for 30 min. Tumor incidence at incision sites was 33% in rats with an open abdomen, 40% in rats exposed to helium insufflation and 57% in rats exposed to STCO_2_ insufflation [63]. 

This higher incidence of tumor cell implantation is theorized to be associated with the “chimney effect”. First there is mobilization of tumor cells during surgical manipulation and subsequent aerosolization within the pneumoperitoneum. Second, gas leakage through port sites causes aggregation of CO_2_ and aerosolized cells around the port sites leading to attachment of the neoplastic cells [64,65]. 

Given this concern alternatives to STCO_2_ have been investigated. Alternative gases such as helium have been evaluated to have reduced incidence of port site metastasis, however the increase in IAP induced with a pneumoperitoneum stretches the abdominal wall, releasing inflammatory mediators, which may also predispose to tumor cell implantation [65]. 

In an in vitro study by Cai et al. colon carcinoma cell lines were exposed to WHCO_2_ at 42–44 °C, >95% humidity at 15 mm Hg for 3 h and cell viability, apoptosis, migration and adhesion were assessed [67]. WHCO_2_ inhibited cell viability to 35–50% and increased apoptosis from 8% to 45–60% [67]. Cell migration was inhibited in a temperature dependent manner and a temperature dependent down-regulation of the adhesion biomarker matrix-metalloproteinase-2 was reported [67]. The authors concluded that WHCO_2_ had cytotoxic effects on carcinoma cells leading to decreased invasive and metastatic potential, while STCO_2_ had similar cell growth patterns as the control incubations [67]. 

There is only one incidence of port site metastasis reported in the veterinary literature, where incisional metastasis of mesothelioma was confirmed at a thoracoscopic port site following pericardial window creation [64]. Even if the incidence is low, given the risk for significant clinical impact, curative laparoscopic procedures for malignant conditions are controversial and should be approached with caution [68]. 

Investigation into the conservation of mesothelial cells in canine pneumoperitoneum and its impact on the risk of port site metastasis is an area of important and active research. Current recommendations for veterinary oncologic patients undergoing laparoscopy with STCO_2_ insufflation is to practice careful dissection to avoid tumor penetration and to use a specimen retrieval bag to minimize metastatic spread [69,70]. 

### 3.5. Pain

Laparoscopic procedures in dogs reduce postoperative pain, surgical stress, and improves postoperative recovery compared to traditional open approaches [13,17,71]. Davidson et al. evaluated postoperative pain in dogs undergoing laparoscopic or traditional ovariohysterectomy using a combination of the Melbourne pain score and subjective behavior evaluation revealing less post-operative pain in dogs undergoing the laparoscopic surgery [71]. Similarly Case et al. used a simple descriptive scale, visual analog scale and von Frey filament to determine postoperative pain scores in cats undergoing laparoscopic or traditional ovariohysterectomy [72]. These authors reported a significant reduction in pain scores in cats undergoing the laparoscopic surgery [72]. 

Postoperative pain following a laparoscopic procedure while less severe than for an open laparotomy, is still a source of surgical stress [73]. A major component of postoperative pain in human laparoscopy is associated with abdominal distension and CO_2_ insufflation, due to irritation and stretching of the phrenic nerves causing referred shoulder pain [74]. This type of neurologic discomfort is not reported in the veterinary literature, however abdominal wall lift (AWL) laparoscopy has been suggested as an alternative modality to minimize this source of pain [75,76]. In a study by Fransson et al. the degree of pain experienced in both STCO_2_ and AWL was similar, suggesting that stretching of the peritoneal tissues is responsible for laparoscopic postoperative discomfort [75]. 

The current veterinary literature supports STCO_2_ insufflation as AWL was unable to demonstrate a significant different in post-operative pain [75]. 

### 3.6. Alternatives to STCO_2_ Insufflation

With detrimental effects such as cardiorespiratory alterations, evaporative hypothermia, peritoneal desquamation, port site metastasis and pain associated with STCO_2_ insufflation, alternative methods for creating a working space within the peritoneal cavity have been developed.

Inert gases such as helium and nitrous oxide have been used to create a pneumoperitoneum without the detrimental effects of systemically or locally absorbed CO_2_ [7,9,77]. Helium insufflation has been proposed to produce less cardiorespiratory and intra-peritoneal immunologic changes compared to STCO_2_ [9,77]. In 20 human patients who underwent laparoscopic cholecystectomy, helium pneumoperitoneum was found to preserve pH and PaCO_2_, however protracted subcutaneous emphysema was noted in 15% of patients, taking several weeks to resolve [7]. This is because helium is less soluble than CO_2_ and more serious potential complications include an increased risk of fatal venous gas embolism [9]. 

Nitrous oxide was the preferred gas in the 1970s but its use for insufflation during laparoscopy declined due to concerns for combustibility limiting its use with electrocautery devices [9]. 

While the use of alternative gases may combat the concern of systemic acidosis and hypercapnia, the cardiorespiratory effects seen in pneumoperitoneum are primarily related to increases in the IAP [78]. Gasless laparoscopy or abdominal wall lift laparoscopy (AWL) has been suggested as an alternative to minimize the cardiorespiratory concerns and has also been hypothesized to reduce perioperative hypothermia [22,76,78]. AWL laparoscopy is performed by inserting a spiraled hook into a small incision in the abdominal wall. This hook is then suspending from the operating room ceiling. This creates a “tent like” effect distending the abdomen from one point, limiting compression on the diaphragm, and therefore minimizing effects on pulmonary compliance. In 2013 Watkins et al. reported a 14% reduction in thoracic volume with STCO_2_ laparoscopy compared to 1% with AWL conditions [79]. The same authors also reported a 55% reduction in working space with AWL compared to STCO_2_ [79]. Despite the proposed benefits Fransson et al. (2014) failed to find any significant difference in cardiorespiratory variables in dogs undergoing AWL laparoscopy compared to STCO_2_ insufflation for ovariohysterectomy [75]. The authors of this study did not find it ethically justifiable to place invasive monitoring equipment, therefore did not measure cardiac output, stroke volume, systemic vascular resistance or pulmonary compliance. The lack of these data limits the ability to interpret the cardiorespiratory findings [75]. 

In conclusion these alternative techniques to maintain a working space have failed to demonstrate any benefit over standard CO_2_ insufflation.

## 4. Discussion

In a literature review of CO_2_ insufflation used for laparoscopy in human and veterinary medicine identified several detrimental effects, including cardiorespiratory depression, acidosis, hypothermia, peritoneal damage, incisional metastasis and postoperative pain [21,23,25]. Of particular interest due to its wide spread implications is the potential for exacerbation of perioperative hypothermia [35]. Hypothermia is thought to develop in STCO_2_ due to evaporative heat lost in saturating insufflated CO_2_ particles, [80] but was not prevented by use of WHCO_2_ [28]. Perioperative hypothermia increases the risk for prolonged recovery, coagulopathies, delayed wound healing and organ dysfunction [36,40]. The cardiorespiratory considerations during laparoscopy are also of importance [25]. A reduction in venous return due to the collapse of the inferior vena cava and changes in vascular resistance secondary to hypercapnia is a considerable dilemma in humans owing to the potential implications to cardiac output [27]. In most studies it appears that while a reduction in stroke volume is evident cardiac output is preserved due to the cardiostimulatory effects of CO_2_ and subsequent tachycardia [10,24,25,28]. Additionally most cardiorespiratory alterations appear transient and while of consideration appear well compensated for in the healthy patient when using the recommended intra-abdominal pressures of 10 mm Hg for dogs and 8 mm Hg for cats [10,24,26,29]. 

## 5. Conclusions

While laparoscopy demonstrates a reduced inflammatory and pain response compared to a laparotomy, efforts are being made to minimize these effects further. Widespread peritoneal cell damage is reported with STCO_2_ which may predispose to discomfort, adhesion formation or tumor cell implantation [53,61]. 

In an effort to minimize these detrimental effects alternative pneumoperitoneal environments have been evaluated, however, to date insufflation with STCO_2_ at IAP of 8–10 mm Hg appears to be the safest and most effective way to create a working space within the abdomen during laparoscopy in small animals.

It remains important to diligently monitor the degree of hypothermia, post-operative discomfort and cardiorespiratory alterations seen with an increased IAP and STCO_2_ pneumoperitoneum.

## Figures and Tables

**Table 1:** Complications associated with perioperative hypothermia [44].

Complications	Reference
Organ dysfunction	Lenhardt, Marker, Goll et al. 1997 Frank, Fleisher, Breslow et al. 1997 Armstrong & Roberts 2005 Clark-Price 2015
Coagulopathy/Increased transfusion requirements	Schmeid, Kurz, Sessler et al. 1996 Lenhardt, Marker, Goll et al. 1997 Kettner, Sitzwohl, Zimpfer et al. 2003 Rundgren & Engstrom 2008 Clark-Price 2015
Impaired humoral and cellular immunity	Kurz, Sessler, Lenhardt 1996 Lenhardt, Marker, Goll et al. 1997 Beilin, Shavit, Razumovsky et al. 1998 Armstrong & Roberts 2005 Beal, Brown, Shofer 2009 Clark-Price 2015
Prolonged recovery	Kurz, Sessler, Lenhardt 1996 Lenhardt, Marker, Goll et al. 1997 Pottie, Dart, Perkins et al. 2007 Clark-Price 2015
